# Validation of the postoperative prognostication tool PREDICT version 2.2 and 3.0 using data from the National cancer center hospital in Japan

**DOI:** 10.1007/s12282-026-01823-w

**Published:** 2026-01-19

**Authors:** Hiromi Hashiguchi, Nobuji Kouno, Masaaki Komatsu, Sho Shiino, Naoto Takehara, Katsuji Takeda, Satoshi Takahashi, Yi-Wen Hsiao, Takeshi Murata, Kazutaka Obama, Shin Takayama, Paul D. P. Pharoah, Ryuji Hamamoto

**Affiliations:** 1 Department of Breast Surgery, National Cancer Center Hospital, Tokyo, Japan; 2 Division of Medical AI Research and Development, National Cancer Center Research Institute, Tokyo, 104-0045 Japan; 3 AI Medical Engineering Team, RIKEN Center for Advanced Intelligence Project, Tokyo, Japan; 4 Department of Surgery, Graduate School of Medicine, Kyoto University, Kyoto, Japan; 5 Department of Breast Surgery, Shin-Matsudo Central General Hospital, Chiba, Japan; 6 Department of Computational Biomedicine, Cedars-Sinai Medical Center, Los Angeles, CA USA

**Keywords:** PREDICT, Japanese breast cancer, Validation, Calibration, Discrimination

## Abstract

**Background:**

PREDICT is a prognostic tool developed in the United Kingdom to estimate postoperative overall survival (OS) and the additional benefits of adjuvant therapies in patients with breast cancer. It has been validated in various international cohorts and continuously updated with the inclusion of new variables and model retraining. However, their efficacy in the Japanese population remains unclear. We aimed to evaluate the generalizability of PREDICT versions 2.2 (v2.2) and 3.0 (v3.0) using data from the National Cancer Center Hospital in Japan, a high-volume cancer center.

**Methods:**

We analyzed a retrospective cohort (2006–2016) including 2,980 cases of postoperative breast cancer. We calculated survival predictions using both v2.2 and v3.0, and compared them with the Kaplan–Meier-estimated survival probabilities using a calibration plot. Additionally, we performed a time-dependent receiver operating characteristic (ROC) curve analysis for v2.2 and v3.0.

**Results:**

Both models tended to underestimate survival in our cohort, whereas v3.0 showed improved calibration compared to v2.2, for 5- and 10-year OS. Both v2.2 and v3.0 maintained good discriminative performance throughout 10 years, with values under the ROC curve generally above 0.80.

**Conclusions:**

Despite these differences, both versions demonstrated satisfactory performance, suggesting that they can be generalized for Japanese patients with postoperative breast cancer. Notably, v3.0, given its improved calibration, might be more suitable for supporting shared decision making. The model’s performance in predicting 5-year OS supports its generalizability, whereas 10-year projections warrant caution due to limited follow-up. Overall, this study demonstrates that PREDICT is a valuable prognostic tool for Japanese patients with breast cancer.

**Supplementary Information:**

The online version contains supplementary material available at 10.1007/s12282-026-01823-w.

## Introduction

Breast cancer continues to be a significant global health issue with increasing incidence and mortality rates around the world. Breast cancer is the most commonly diagnosed cancer and the most frequent cause of cancer-related deaths [1]. Furthermore, this trend is expected to continue, with three million people predicted to be newly diagnosed and one million people predicted to die by 2040 [2]. Similarly, in Japan, breast cancer has the highest incidence and third-highest mortality among all cancers [3]. The treatment landscape for breast cancer has undergone significant diversification [4]. The need for personalized medicine is increasing owing to the aging population and varied health histories and lifestyles of patients [5]. As treatment options expand, the role of visualization tools in assessing treatment efficacy for each patient has become crucial in guiding postoperative decisions, such as PREDICT [6], Adjuvant! Online [7], and CancerMath [8]. These tools not only aid clinicians in evaluating the potential benefits of various therapies but also empower patients by providing clearer insights into their treatment pathways, ultimately enhancing shared decision-making in breast cancer care.

PREDICT is freely available online and is widely recognized for its ability to visualize additional therapeutic effects and overall survival (OS) on postoperative breast cancer. PREDICT was first developed in 2010, drawing on data from over 5,000 women undergoing surgery for early breast cancer between 1999 and 2003 in the United Kingdom (UK), mainly by the Cambridge University group [6]. Since its launch, PREDICT has been repeatedly validated using data cohorts in Western countries, including UK [9, 10], the United States (US) [11], Canada [12], the Netherlands [13, 14] and Spain [15]. PREDICT has been updated several times to enhance its predictive accuracy and clinical relevance [16]. Notably, the step-by-step inclusion of additional prognostic factors have been made so far: HER2/ERBB2 status [17], Ki-67 expression [18] and progesterone receptor (PR) status [19], has improved its ability to estimate treatment benefits and OS. PREDICT version 3.0 (v3.0) has been publicly released recently, with the update of radiation therapy added to the treatment options [20]. PREDICT v3.0 was validated using the Surveillance, Epidemiology, and End Results (SEER), a large US epidemiological dataset [21]; it showed accurate long-term survival estimates [22]. Compared with v2.2, PREDICT v3.0, showed improved calibration and slightly better discrimination [22].

Recently, PREDICT has been subjected to validation studies across various Asian countries, providing insights into its applicability and accuracy for patients with breast cancer in these regions. In a retrospective cohort of 2,783 Indian women with non-metastatic, HER2-negative operable breast cancer, v2.2 underestimated the 5-year OS (observed: 94.8% vs. predicted: 90.0%), with notable discrepancies in younger patients, high-grade tumors, node-positive disease, and triple-negative subgroups [23]. In a Malaysian cohort of 355 patients with breast cancer, PREDICT v2.2 accurately estimated the 5-year OS (observed: 75.2% vs. predicted: 75.8%), demonstrating good calibration and discrimination [24]. Furthermore, in a large Chinese cohort, PREDICT v3.0 accurately estimated 5-year OS and showed significantly improved calibration compared to PREDICT v2.2, especially in estrogen receptor (ER)-negative patients [25]. In Japan, PREDICT v2.2 reliably estimated 5- and 10-year OS among operative breast cancer cases, though it underestimated long-term outcomes in subgroups such as patients aged ≥ 65, those with grade 3 or Luminal A tumors, and those receiving endocrine therapy alone or no systemic therapy [26].

Given the diverse backgrounds of patients with breast cancer, PREDICT is a valuable tool for facilitating personalized medicine. Although studies support its general applicability in Asian populations, extrapolation of the recent PREDICT v3.0 to Japanese female patients with breast cancer has not yet been sufficiently verified. No study to date has directly compared the prognostic performance of PREDICT v2.2 and v3.0 within the same Japanese cohort. In this study, we validated PREDICT v2.2 and v3.0, in a cohort of nearly 3,000 women undergoing surgeries for early breast cancer by directly comparing the calibration and discrimination of the two versions under identical clinical conditions, leading to findings contributing to personalized medicine for Japanese patients with breast cancer.

## Patients and methods

### Patients

We collected data from 3,000 Japanese women with primary breast cancer who underwent surgery at the National Cancer Center Hospital between 2006 and 2016. Patients were included in the study if they had complete clinicopathological data and details of postoperative therapy available. The study excluded cases of male breast cancer, bilateral breast cancer (including both simultaneous and asynchronous occurrences), Stage IV disease at initial diagnosis, and noninvasive carcinoma. Patients who received neoadjuvant chemotherapy or hormone therapy before surgery were excluded. Patients aged ≤ 24 or ≥ 86 years were excluded. Staging was based on the 8th edition of the Union for International Cancer Control (UICC) classification system.

This study was approved by the Institutional Review Board of the National Cancer Center (protocol code: 2016 − 496).

## Description of PREDICT

### PREDICT v2.2

Retrieved from https://breast.predict.cam, PREDICT v2.2 integrates a comprehensive range of clinicopathological and treatment-related variables to project OS at 5, 10, and 15 years post-surgery, with or without adjuvant therapies.

Patient-specific inputs included age at diagnosis (25–85 years), menopausal status (pre-, postmenopausal, or unknown), and ER status (positive or negative). HER2/ERRB2 and Ki-67 statuses were recorded as positive, negative, or unknown, with “positive” defined as > 10% in the case of Ki-67 following official specifications. Tumor-related variables included histological grade (1, 2, or 3), tumor size in millimeters (recorded prior to any neoadjuvant therapy), and detection mode (screen-detected, symptomatic, or unknown). Patients diagnosed with ductal carcinoma in situ or lobular carcinoma in situ were excluded because PREDICT is not applicable to noninvasive breast cancers. Nodal involvement was characterized by the number of positive axillary lymph nodes and presence of micrometastases (yes, no, or unknown).

Treatment inputs for PREDICT v2.2 reflected the actual postoperative therapies administered to each patient. Therapeutic scenarios were simulated using available treatment options within the model, including hormone therapy (none, 5 years, or 10 years), chemotherapy (none, second-, or third-generation regimens), and targeted therapy with trastuzumab (available for HER2-positive patients). Bisphosphonates were used in this study.

PREDICT v2.2 provides estimates of 5-, 10-, and 15-year OS, as well as the additional survival benefit of adjuvant treatments, including hormone therapy, chemotherapy, anti-HER2 therapy, and bisphosphonates, compared with surgery alone. The model also visualizes the survival probability of an individual patient over time.

### PREDICT v3.0

PREDICT v3.0 (https://breast.v3.predict.cam), released in 2024, is a substantial update to the original model architecture [20]. Most notably, the underlying Cox proportional hazards models were retrained using a contemporary UK cohort comprising over 35,000 women diagnosed with breast cancer between 2000 and 2017. This retraining involved recalibration of both the baseline cumulative hazard functions and regression coefficients. In addition to the variables previously incorporated in v2.2, v3.0 explicitly includes the year of diagnosis as a covariate to account for improvements in survival over time. Furthermore, treatment-related variables in v3.0, now incorporate not only the benefits but also the potential harms of local and systemic therapies, specifically the increased risk of non-breast cancer mortality associated with radiotherapy. While the structure of the input variables remained largely consistent with v2.2, the computational backend and risk estimates were fundamentally updated, resulting in improved calibration for external validation [22].

Compared to v2.2, PREDICT v3.0 introduces additional input variables (year of diagnosis, smoking status, and progesterone receptor status) and incorporates the treatment effects of radiotherapy and mean heart dose [20].

### Handling missing data for PREDICT input

For the variable bisphosphonates, which are included in both PREDICT v2.2 and v3.0, treatment records are entirely absent from our dataset; therefore, this variable was uniformly coded as “No.” For variables such as Ki-67 in v2.2 and v3.0, where “unknown” can be selected as the default option when using the model, missing values were assigned as “unknown.” The variable smoking status, newly introduced in v3.0, was completely overlooked in our dataset; therefore, we coded it as “No” for all cases. Regarding the mean heart dose in Gy for patients who underwent radiotherapy, also equipped in v3.0, no corresponding data were available. Then following the instructions on the official website, we imputed this variable as “0” for right-sided tumors and “2” for left-sided tumors.

### PREDICT output calculation

Survival probabilities were calculated using the PREDICT model implemented as R packages available from public GitHub repositories; v2.2 was cloned from the Winton Centre’s repository (https://github.com/WintonCentre/predict-v21-r), and v3.0 was obtained from the Peng’s lab (https://github.com/pengpclab/PREDICTv3). Survival probability predictions from v2.2 and v3.0, were computed using R scripts developed for this analysis. For each patient, the predicted survival probabilities were calculated annually from 1 to 10 years postoperatively.

### Model performance evaluation for calibration

To evaluate the calibration, we calculated survival probabilities using the Kaplan–Meier (KM) method at both the cohort and subclass levels. The KM method is a nonparametric univariate survival analysis technique that estimates time-to-event probabilities while accounting for censored observations [27]. We defined the survival probabilities estimated using the KM method as the observed OS. OS was defined as being alive at a specific time point, with death from any cause considered an event. Patients who were alive at the last follow-up were censored at that date. Compared to the observed OS, predicted survival probabilities were calculated using PREDICT v2.2 and v3.0, applied to each sample in the entire cohort at baseline. For each patient, the survival probabilities for each year, up to 10 years postoperatively were calculated. The predicted survival probabilities of the entire baseline and subclass cohorts were derived by averaging the individual estimates. Predicted survival probabilities were defined as predicted OS.

Because both the KM estimates and survival probabilities predicted by PREDICT represent expected values rather than observed counts, formal statistical hypothesis testing, such as the chi-square test, was not applied to their comparison. Instead, model calibration was assessed visually using calibration plots in which the observed 5- and 10-year OS were plotted against the corresponding predicted OS.

For the calibration plots for postoperative 5 and 10 years, the patients were stratified into quintiles based on the predicted OS at the target year. The mean predicted OS was calculated for each quintile. The observed OS and confidence intervals (CI) were calculated and plotted within each quintile.

### Model performance evaluation for discrimination

Model discrimination was evaluated using time-dependent receiver operating characteristic (ROC) curves and the corresponding area under the curve (AUC), which assessed the model’s ability to distinguish between individuals who experienced death and those who did not at each time point. Time-dependent ROC curve analysis evaluates the predictive accuracy of a model at specific time points, accounting for changes in model performance over time in longitudinal studies [28]. Time-dependent ROC curves were constructed, and the corresponding AUCs were calculated using the inverse probability of the censoring weighting (IPCW) method to account for censored observations. We generated 1,000 bootstrap samples for the ROC curve for each year. The distribution of AUCs across bootstrap samples was used to estimate the 95% CIs, allowing us to evaluate the discriminative ability of PREDICT over time.

## Results

### Baseline characteristics stratified by Estrogen receptor status

The patient demographics, tumor features, and treatment information are presented in Table 1, stratified by ER status. Twenty patients were excluded because the applicable age range was 25–85 years in the PREDICT model. The total population comprised 2,980 patients with invasive breast cancer, including 2,464 (82.7%) with ER-positive tumors and 516 (17.3%) with ER-negative tumors. The median age at diagnosis was 55 years for all patients, 54 years for ER-positive cases, and 59 years for ER-negative cases. The median follow-up time was 7.0 years for both groups. The total person-years were 21,581, with 17,856 and 3,724 person-years in the ER-positive and ER-negative groups, respectively.

**Table 1:** Patient demographics stratified by ER status

	Total	ER positive	ER negative
Patients, *n*	2,980	2,464	516
Age at diagnosis, median (IQR)	55 (46–65)	54 (45–64)	59 (50–67)
Follow-up time, median (IQR)	7.0 (5.8–9.2)	7.0 (5.8–9.2)	7.0 (5.7–9.5)
Person-years	21,581	17,856	3,724
Post-menopausal			
No, *n* (%)	1,271 (42.7)	1,128 (45.8)	143 (27.7)
Yes, *n* (%)	1,709 (57.3)	1,336 (54.2)	373 (72.3)
PR status			
Negative, *n* (%)	809 (27.1)	326 (13.2)	483 (93.6)
Positive, *n* (%)	2,171 (72.9)	2,138 (86.8)	33 (6.4)
HER2 status			
Negative, *n* (%)	2,586 (86.8)	2,252 (91.4)	334 (64.7)
Positive, *n* (%)	394 (13.2)	212 (8.6)	182 (35.3)
Ki-67 status			
Negative, *n* (%)	405 (13.6)	392 (15.9)	13 (2.5)
Positive, *n* (%)	1,245 (41.8)	1,027 (41.7)	218 (42.2)
Unknown, *n* (%)	1,330 (44.6)	1,045 (42.4)	285 (55.3)
Tumor size, median (IQR), mm	17 (11–25)	17 (11–25)	18 (11–25)
Tumor grade			
1, *n* (%)	582 (19.5)	559 (22.7)	23 (4.5)
2, *n* (%)	1,410 (47.3)	1,306 (53.0)	104 (20.2)
3, *n* (%)	988 (33.2)	599 (24.3)	389 (75.3)
Detection			
Screening, *n* (%)	1,299 (43.6)	1,108 (45.0)	191 (37.0)
Symptomatic, *n* (%)	1,681 (56.4)	1,356 (55.0)	325 (63.0)
Positive nodes, median (min - max)	0 (0–40)	0 (0–40)	0 (0–27)
Surgery			
Mastectomy, *n* (%)	1,362 (45.7)	1,077 (43.7)	285 (55.2)
Partial, *n* (%)	1,618 (54.3)	1,387 (56.3)	231 (44.8)
Radiotherapy			
No, *n* (%)	1,296 (43.5)	1,033 (41.9)	263 (51.0)
Yes, *n* (%)	1,684 (56.5)	1,431 (58.1)	253 (49.0)
Chemotherapy			
No, *n* (%)	1,807 (60.6)	1,630 (66.1)	177 (34.3)
2nd gen, *n* (%)	673 (22.6)	485 (19.7)	188 (36.4)
3rd gen, *n* (%)	500 (16.8)	349 (14.2)	151 (29.3)
pStage (UICC 8th)			
I, *n* (%)	1,495 (50.2)	1,251 (50.8)	244 (47.3)
II, *n* (%)	1,029 (34.5)	852 (34.6)	177 (34.3)
III, *n* (%)	456 (15.3)	361 (14.6)	95 (18.4)

Although the PREDICT model permits unknown entries for postmenopausal status, progesterone receptor status, and HER2/ERBB2 status, all these variables were complete in our dataset. Ki-67 status was unknown in 44.6% of patients.

### Model calibration

Table 2 summarizes the observed and predicted 5-year OS. In the ER-positive group, the observed 5-year OS was 98.8%, whereas v2.2 and v3.0 predicted 91.8% and 95.5%, respectively. Similarly, in the ER-negative group, the observed OS was 95.8%, compared to 77.4% by v2.2 and 86.9% for v3.0. Across most stratified subgroups, both versions of PREDICT tended to underestimate survival, though v3.0 showed consistently improved alignment with observed outcomes.

**Table 2:** Observed 5-year OS and predicted OS by PREDICT v2.2 and v3.0, stratified by ER status

Characteristic	Total	ER positive				ER negative			
		*n*	v2.2 (%)	v3.0 (%)	Obs (%)	*n*	v2.2 (%)	v3.0 (%)	Obs (%)
Patients, *n*	2,980	2,464	91.8	95.5	98.8	516	77.4	86.9	95.8
Age at diagnosis									
-39	252	212	92.8	97	96.3	40	80.4	85.9	100
40–49	832	753	95.2	97.9	99.4	79	81.3	90.6	94.7
50–64	1,116	883	93.0	96.4	99.3	233	79.7	89.8	97.3
65-	780	616	85.8	90.6	98.2	164	71.6	81.3	93.2
Post-menopausal									
No	1,271	1,128	94.5	97.6	98.7	143	80.5	89.0	96.3
Yes	1,709	1,336	89.6	93.7	98.9	373	76.2	86.1	95.6
PR status									
Negative	809	326	89.9	93	97.7	483	77.7	86.9	95.8
Positive	2,171	2,138	92.1	95.8	99.0	33	73.0	86.9	96.9
HER2 status									
Negative	2,586	2,252	92.0	95.6	98.9	334	77.5	86.7	95.2
Positive	394	212	90.1	94.2	97.9	182	77.2	87.3	97.0
Ki-67 status									
Negative	405	392	94.3	97.0	99.7	13	77.0	88.4	100
Positive	1,245	1,027	91.2	95.6	98.6	218	78.6	88.7	95.0
Unknown	1,330	1,045	91.5	94.8	98.7	285	76.5	85.5	96.2
Tumor size									
< 10 mm	593	476	95.8	97.4	99.6	117	85.5	95.1	98.2
10 ≤ and < 20 mm	1,152	984	93.4	96.2	99.6	168	80.3	89.3	97.5
20 ≤ and < 30 mm	701	562	90.8	94.8	98.7	139	77.2	86.1	94.6
30 ≤ and < 40 mm	275	229	88.7	93.8	97.5	46	69.4	78.0	95.1
40 ≤ and < 50 mm	113	94	86.8	92.8	96.5	19	65.1	74.5	88.5
50 mm ≤	146	119	78.0	89.7	94.7	27	47.3	64.2	87.9
Tumor grade									
1	582	559	95.0	97.1	100	23	88.9	92.8	90.5
2	1,410	1,306	92.5	95.7	99.5	104	77.5	89.6	99.0
3	988	599	87.5	93.5	96.2	389	76.7	85.8	95.3
Detection									
Screening	1,299	1,108	93.9	96.7	99.4	191	79.9	90.6	96.2
Symptomatic	1,681	1,356	90.2	94.4	98.3	325	75.9	84.7	95.6
Positive nodes									
0	2,090	1,720	93.7	96.3	99.4	370	82.1	90.3	97.7
1	404	348	91.6	95.4	98.4	56	75.9	85.1	96.2
2–4	311	256	88.8	94.0	98.3	55	67.8	80.0	96.0
5–9	123	103	79.5	90.3	95.8	20	51.2	70.2	69.3
10-	52	37	64.2	82.6	86.0	15	38.4	57.2	85.1
Surgery									
Mastectomy	1,362	1,077	90.4	94.6	98.3	285	75.0	84.8	94.6
Partial	1,618	1,387	93.0	96.1	99.2	231	80.4	89.5	97.3
Radiotherapy									
No	1,296	1,033	91.9	95.1	98.8	263	78.4	86.9	96.3
Yes	1,684	1,431	91.8	95.7	98.8	253	76.3	86.9	95.4
Hormone therapy									
No	769	294	90.0	94.2	97.6	475	77.4	86.8	95.7
Yes	2,211	2,170	92.1	95.6	99.0	41	77.3	88.3	97.2
Trastuzumab									
No	2,716	2,312	92.0	95.5	98.8	404	77.4	86.9	95.2
Yes	264	152	89.9	94.6	98.6	112	77.3	86.8	98.1
Chemotherapy									
No	1,807	1,630	92.7	95.7	99.3	177	77.3	87.0	95.6
2nd gen	673	485	92.1	95.7	98.5	188	80.1	88.5	96.1
3rd gen	500	349	87.4	94.1	97.3	151	74.1	84.9	95.7
pStage (UICC 8th)									
I	1,495	1,251	94.4	96.7	99.7	244	83.6	92.3	98.7
II	1,029	852	91.2	95.0	98.6	177	77.0	85.3	95.1
III	456	361	84.6	92.4	96.5	95	62.1	76.0	89.7

ER, estrogen receptor; v2.2, PREDICT version 3.0; v3.0, PREDICT version 3.0; Obs, observed survival probability by Kaplan-Meier method;

Table S1 shows the observed and predicted 10-year OS rates. Among ER-positive patients, the observed OS at 10 years was 96.0%, whereas for v2.2 and v3.0, it was estimated to be 80.5% and 87.9%, respectively. In the ER-negative group, the observed OS was 89.4%, compared to 66.3% predicted by v2.2 and 77.3% predicted by v3.0. Both models underestimated the OS across most subgroups, but v3.0 consistently demonstrated closer agreement with the observed OS, indicating improved calibration over an extended time horizon.

**Fig. 1:**
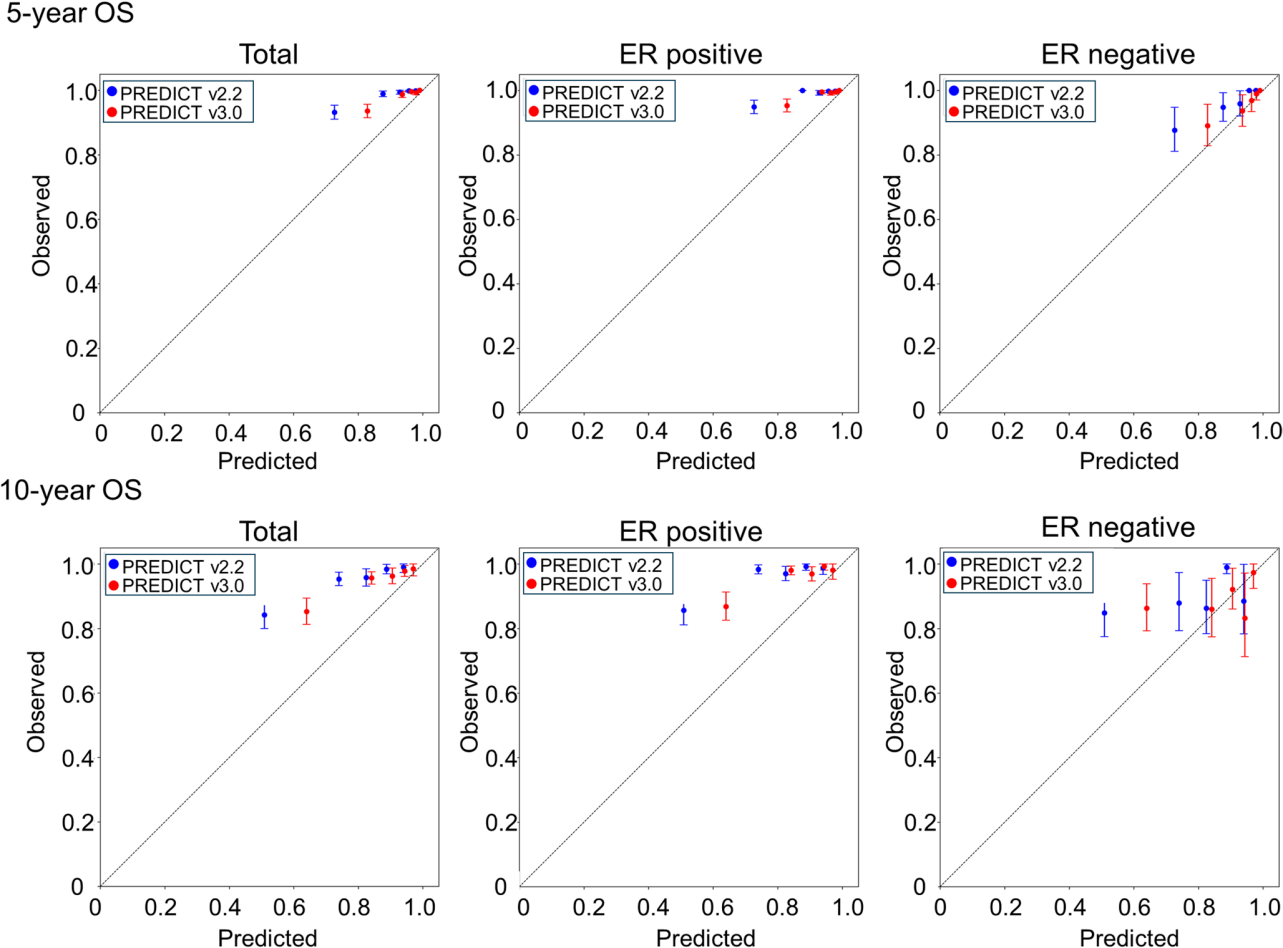
Calibration of PREDICT v2.2 and v3.0 for OS in Japanese patients with breast cancer. Observed versus predicted OS at 5 years (top panels) and 10 years (bottom panels) for the total cohort, ER-positive, and ER-negative subgroups. Predictions from PREDICT v2.2 (blue) and v3.0 (red) are plotted against the observed OS with 95% CI. The dot line represents perfect calibration. ER, estrogen receptor; OS, overall survival; CI, confidence interval

Figure 1 shows calibration plots of the observed 5- and 10-year OS and predicted OS calculated by v2.2 and v3.0, stratified by ER status. The patients were grouped into quintiles based on their predicted OS. Across all groups and time points, both models tended to underestimate survival, with v3.0 showing consistently better alignment with the observed OS. Improvements in the calibration were particularly evident in the total and ER-positive cohorts. In the ER-negative group, variability was greater, but v3.0 still demonstrated a relatively better performance than v2.2. These graphical results are consistent with Table 2 and Table S1, supporting the improved calibration of the v3.0.

In the ER-negative group, as shown in Table S1, the Ki-67-negative subgroup had no patients at risk at the 10-year time point due to censoring. Consequently, the observed 10-year OS could not be estimated using the KM method, and the corresponding cell was left blank.

### Model discrimination for annual OS with time-dependent ROC

**Fig. 2:**
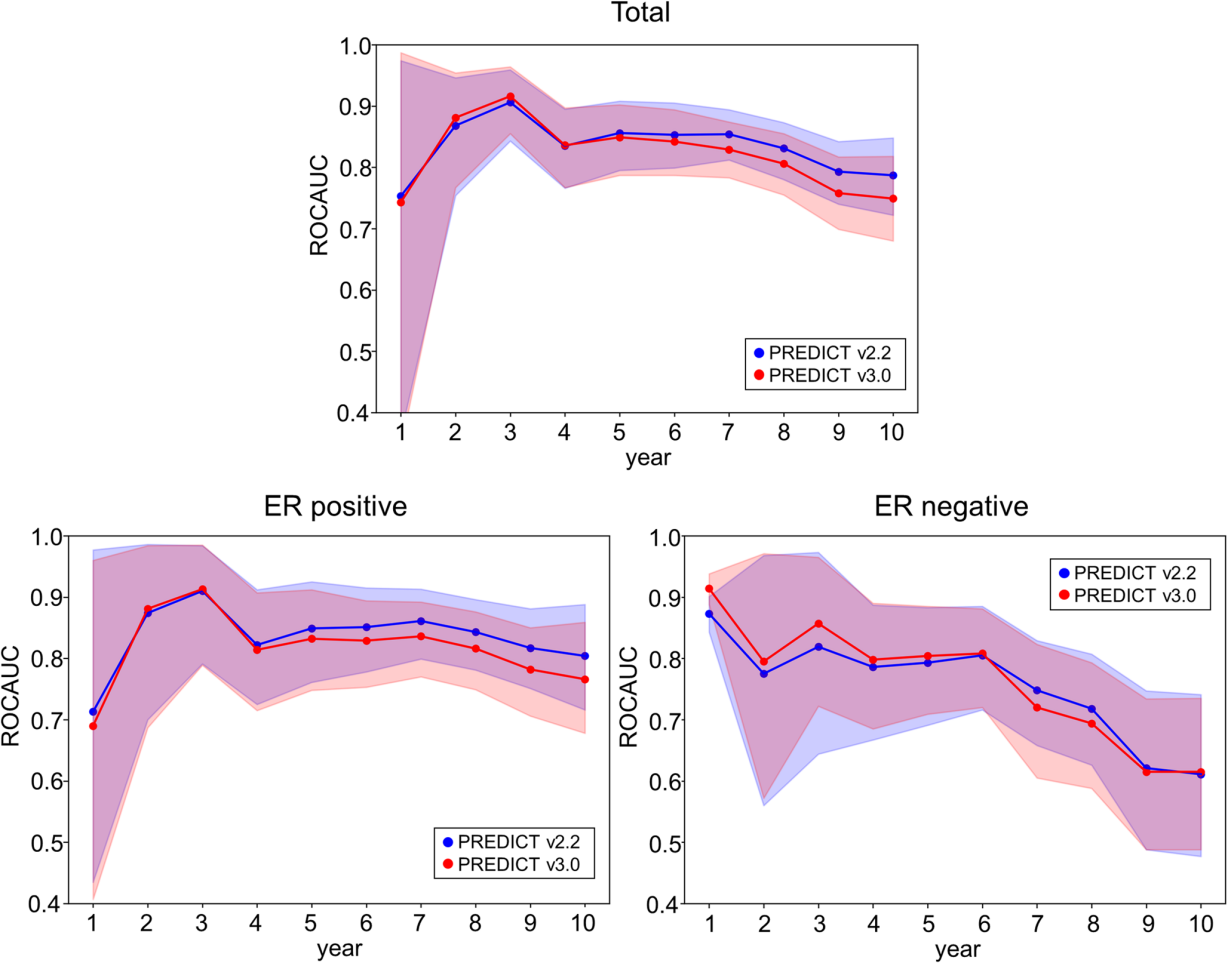
Time-dependent ROC of PREDICT v2.2 and v3.0 for observed OS in Japanese breast cancer patients up to 10 years. From the top left panel and counterclockwise: total cohort, ER-positive, and ER-negative subgroups. PREDICT v2.2 (blue) and v3.0 (red) are shown with 95% CIs. ER, estrogen receptor; OS, overall survival; CI, confidence interval

Figure 2 illustrates the AUC values from years 1 to 10 of the time-dependent ROC for the total, ER-positive, and ER-negative cohorts. In the total and ER-positive cohorts, both v2.2 and v3.0 maintained good discriminative performance throughout the 10-year period, with AUCs generally above 0.80. In particular, v2.2 tended to show slightly higher AUCs over time, especially beyond year five. In contrast, the ER-negative group showed more fluctuations and a gradual decline in AUCs for both models after 5 years. Overall, both models demonstrated acceptable short- and mid-term discrimination, with v2.2 showing slightly better consistency in later years.

Figure S1 presents the KM survival curve for the overall cohort, whereas Figure S2 depicts the curves stratified by ER status. The accompanying tables display the number at risk, events, and censored observations for each annual time point. Because the number of censored cases increased markedly after 5 years and exceeded the number of events beyond 10 years, model performance was evaluated only up to the 10-year time point in this study. At the 10-year time point, there were 96 deaths in the overall cohort (60 in the ER-positive group and 36 in the ER-negative group), and 2,316 censored cases (1,940 in the ER-positive group and 376 in the ER-negative group).

## Discussion

In this study, we externally validated the prognostic performance of PREDICT v2.2 and v3.0, in a Japanese breast cancer cohort. Although PREDICT v2.2 performance has been previously evaluated in a Japanese study, to our knowledge, this is the first to validate the calibration and discrimination of PREDICT v3.0 and directly compare both versions within the same Japanese cohort with minimal missing data. Both versions tended to underestimate the 5- and 10-year survival across different patient subgroups, with v3.0 demonstrating consistently improved calibration. Time-dependent ROC analysis indicated that both models maintained acceptable discrimination, especially in ER-positive patients, although v2.2 showed slightly higher AUCs than v3.0 beyond five years.

These findings highlight the tradeoff between improved calibration and slightly reduced discrimination in the updated model. Although both discrimination and calibration are critical for evaluating prediction models, achieving high performance simultaneously is often challenging, because improvements in one may not guarantee adequacy in the other [29]. Calibration reflects how closely the predicted probabilities match the actual outcomes, whereas discrimination measures a model’s ability to distinguish between patients with and without events [30]. Although both are essential for evaluating predictive models, they are statistically independent, and improving one may inadvertently compromise the other, highlighting a critical tradeoff in model optimization [30, 31].

Our validation results raise the question of how PREDICT v2.2 and v3.0 should be optimally utilized in Japanese patients with postoperative breast cancer. The choice between v2.2 and v3.0 may depend on the intended clinical application. While v2.2 demonstrated superior discriminative performance, v3.0 showed improved calibration. In clinical situations where risk stratification is critical, such as identifying patients who may benefit from adjuvant chemotherapy, the higher time-dependent AUC of v2.2 may be more useful, as it better distinguishes high-risk from low-risk individuals. In addition to guiding chemotherapy decisions, the sustained discriminatory ability of v2.2 beyond 5 years may also be informative when considering the extended use of hormone therapy. Reliable and interpretable survival probabilities are essential when discussing treatment options [32]. In this context, v3.0 may be preferable because of its improved calibration, which enables clinicians to communicate individualized prognostic estimates with greater confidence. Thus, the strengths of each version should be considered in the specific clinical context, whether the goal is to support clinician-led risk-based decision-making or to enhance shared decision-making with patients.

When incorporating PREDICT into the Japanese context, it is important to recognize several key differences in breast cancer epidemiology between Japan and Western countries. First, the peak incidence occurs approximately a decade earlier in Japan and East Asia (aged 40–50), whereas it typically peaks in Western populations at 60–70 years [33]. This younger age distribution may have influenced the predictive accuracy and validity of the PREDICT models developed based on Western cohorts. Second, while the incidence and mortality began to decline in the West in the 1990s, both have continued to increase in Japan, likely due to shifting reproductive patterns and lifestyle changes such as delayed childbirth and increased obesity [34]. This divergence suggests that calibration improvements in v3.0 may not fully translate to the Japanese population unless region-specific mortality trends are considered. Third, biological heterogeneity differs; East Asian populations tend to have a higher proportion of luminal B subtypes, TP53 mutations, and more immune-active tumor microenvironments than Western populations [35]. These distinctions may explain some of the reduced discriminative performance observed in ER-negative patients who inherently display greater heterogeneity. Fourth, registry-based comparisons showed that Japanese women and Japanese Americans of Japanese descent have significantly better overall and breast-cancer–specific survival than US women of other ethnicities, even after adjusting for stage and receptor status [36]. This highlights the importance for exercising caution when applying Western-derived prognostic models to Japanese cohorts. As breast cancer epidemiology continues to evolve in Japan, future model updates should consider integrating regional factors.

Although our findings indicate some limitations when applying PREDICT v2.2 and v3.0, to Japanese patients with breast cancer, these models remain globally respected, clinically practical tools that have meaningfully advanced prognostic modeling. PREDICT v3.0, in particular, offers improved calibration. Additionally, research on predictive models that leverage artificial intelligence techniques, such as machine learning and deep learning, has rapidly progressed in recent years, supporting personalized medicine. An AdaBoost-based model integrating routine clinical and laboratory features demonstrated high accuracy and interpretability in predicting breast cancer recurrence, offering a practical decision-support tool for early intervention [37]. The myBeST tool provides a locally validated web-based platform to support individualized 5-year survival estimation for Malaysian women with breast cancer, thereby enhancing risk communication in clinical practice [38]. A deep learning survival model built on clinicopathological data from a large cancer registry showed higher accuracy in predicting mortality risk in triple-negative breast cancer than conventional staging and statistical methods [39]. By integrating domain-specific knowledge with these machine learning techniques, it is possible to develop a unique model that can contribute to personalized medical decisions in Japan.

This study had some limitations. First, some input variables are either entirely or partially missing. Bisphosphonate use and smoking status were not consistently collected across all cases, and missing values were pragmatically imputed as “No.” This approach reflects real-world clinical practice in Japan, where adjuvant bisphosphonate therapy is not routinely prescribed and smoking prevalence among women is relatively low. Similarly, information on the mean heart dose during radiotherapy was unavailable; therefore, it was imputed according to the official guidelines provided on the PREDICT website. This imputation strategy was chosen to ensure consistency with the model specifications. These imputations may have reduced the accuracy of estimated survival probabilities. In addition, approximately half of the patients had an “Unknown” status for Ki-67. Although “Unknown” is an acceptable input option in the model and not technically considered missing, this variable may still have influenced the results. Therefore, the potential impact of missing or imputed variables on the model validation outcomes cannot be entirely excluded [40]. Second, a substantial proportion of censored cases may have influenced the comparison with the predicted outcomes. Of the initial 2,980 patients, approximately 400 and over 2,300 were censored at 5 and 10 years, respectively (Fig. S1 and S2). This high censoring rate limits the number of events available for comparison at later time points. The results near the right end of the KM curve, where few subjects remain under observation owing to censoring, should be interpreted with caution [27]. Third, only a small number of patients with ER-negative tumors were recorded as having undergone hormone therapy. This may reflect historical variations in diagnostic thresholds, the presence of PR positivity or low-level receptor expression, clinical discretion or patient preference, as well as potential data recording errors.

In conclusion, this study externally validated the performance of PREDICT v2.2 and v3.0 within a large cohort of Japanese patients with breast cancer. Both models tended to underestimate survival rates; however, v3.0 demonstrated improved calibration, while v2.2 maintained slightly better discrimination, particularly beyond five years. The 5-year overall survival analysis serves as the primary evaluation of model performance in this study. However, findings regarding 10-year survival should be interpreted as exploratory due to the high proportion of censored cases at later time points. These findings suggest that both versions are applicable to Japanese patients, but their optimal use should be tailored to the clinical context: v2.2 for risk stratification and v3.0 for individualized prognostic communication. Given regional differences in breast cancer epidemiology and treatment practices, future research should focus on developing and validating prediction models specifically optimized for Japanese clinical settings.

## Supplementary Information

Below is the link to the electronic supplementary material.

Supplementary Material 1

## Data Availability

The data used in this study are available from the corresponding author upon request.

## Abbreviations

OS: Overall survival
UK: United Kingdom
US: United States
PR: Progesterone receptor
v3.0: version 3.0
SEER: Surveillance, Epidemiology, and End Results
UICC: Union for International Cancer Control
v2.2: version 2.2
ER: Estrogen receptor
KM: Kaplan-Meier
CI: Confidence interval
ROC: Receiver operating characteristic
AUC: Area under the curve
IPCW: Inverse probability of censoring weighting
