# Supplementary material for: Validation of the postoperative prognostication tool PREDICT version 2.2 and 3.0 using data from the National cancer center hospital in Japan
Source: Breast Cancer. 2026 Jan 19;33(2):415–25. doi: 10.1007/s12282-026-01823-w (PMC12960381; doi:10.1007/s12282-026-01823-w)
Supplement: Supplementary file 1 — Supplementary Material 1 [file 12282_2026_1823_MOESM1_ESM.pdf]

Supplementary information for

Validation of the postoperative prognostication tool PREDICT version 2.2 and 3.0 using data from the National Cancer Center Hospital in Japan

Hiromi Hashiguchi, Nobuji Kouno, Masaaki Komatsu, Sho Shiino, Naoto Takehara, Katsuji Takeda, Satoshi Takahashi, Yi-Wen Hsiao, Takeshi Murata, Kazutaka Obama, Shin Takayama, Paul D.P. Pharoah, Ryuji Hamamoto

The file contains

Supplementary Figs. 1 – 2

Supplementary Table 1

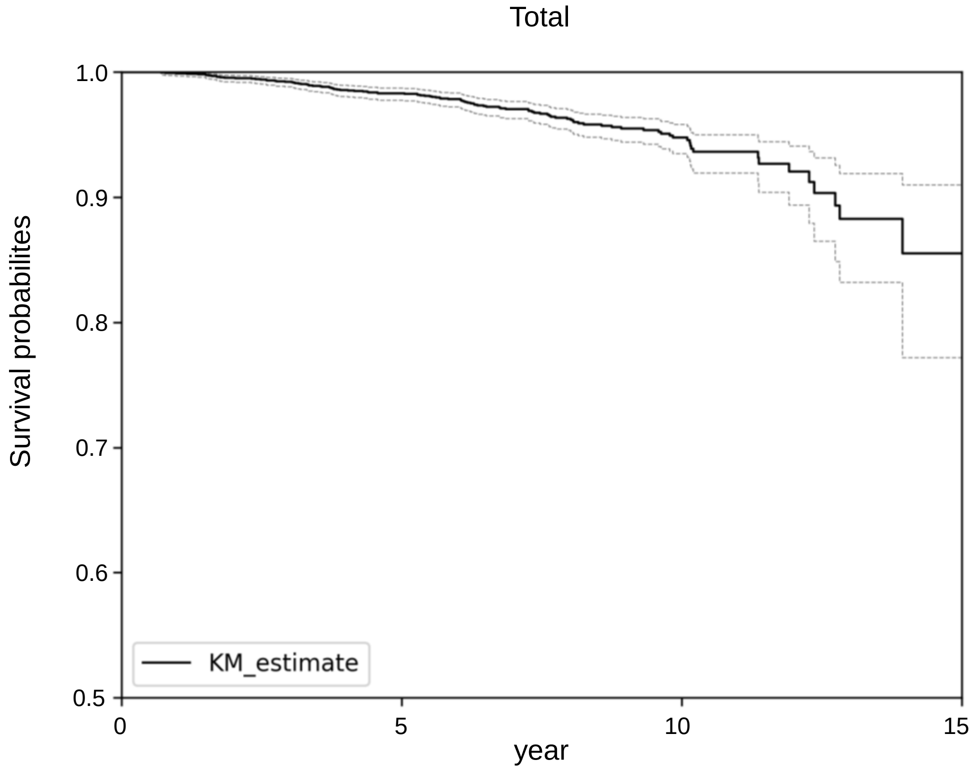

Year	1	2	3	4	5	6	7	8	9	10	11	12	13	14	15
Number at risk	2893	2819	2748	2650	2552	2132	1512	1120	834	568	264	138	73	30	15
Censored	84	148	210	290	381	790	1395	1777	2055	2316	2615	2738	2799	2841	2856
Death	3	13	22	40	47	58	73	83	91	96	101	104	108	109	109

Fig. S1 Kaplan-Meier (KM) curve for the total cohort up to 15 years with 95% confidence intervals (CIs). The table below the plot shows the number at risk, censored cases, and death events at each yearly time point.

ER positive

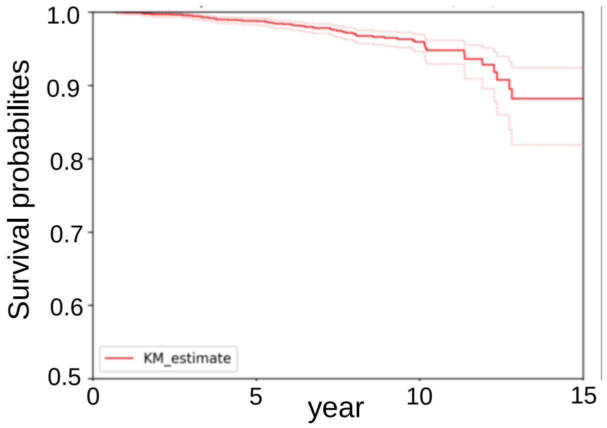

Year	1	2	3	4	5	6	7	8	9	10	11	12	13	14	15
Number at risk	2391	2335	2284	2199	2120	1771	1249	922	681	464	218	115	57	21	13
Censored	71	122	169	242	317	657	1171	1490	1726	1940	2182	2282	2336	2372	2380
Death	2	7	11	23	27	36	44	52	57	60	64	67	71	71	71

ER negative

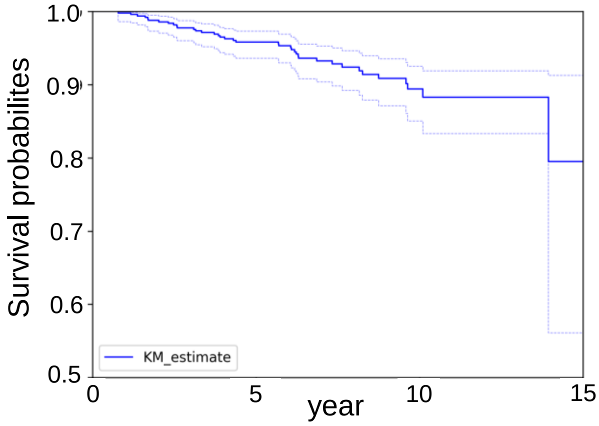

Year	1	2	3	4	5	6	7	8	9	10	11	12	13	14	15
Number at risk	502	484	464	451	432	361	263	198	153	104	46	23	16	9	2
Censored	13	26	41	48	64	133	224	287	329	376	433	456	463	469	476
Death	1	6	11	17	20	22	29	31	34	36	37	37	37	38	38

Fig. S2 KM curves for ER-positive and ER-negative cohorts up to 15 years for patients with ER-positive (top) and ER-negative (bottom) breast cancer with 95% CIs. The tables below the plots present the number at risk, censored cases, and death events at each yearly time point. ER, estrogen receptor; CI, confidence interval.

Table S1 Observed 10-year overall survival (OS) and predicted OS by PREDICT version 2.2 and version 3.0, stratified by estrogen receptor status

		**ER positive**				**ER negative**			
**Characteristic**	**Total**	***n***	**v2.2 (%)**	**v3.0 (%)**	**Obs (%)**	***n***	**v2.2 (%)**	**v3.0 (%)**	**Obs (%)**
**Patients,** *n*	2,980	2,464	80.5	87.9	96.0	516	66.3	77.3	89.4
**Age at diagnosis**									
**-39**	252	212	84.1	92.4	84.7	40	73.6	79.4	84.7
**40-49**	832	753	88.1	94.3	97.4	79	74.6	85.5	91.4
**50-64**	1,116	883	82.8	90.3	98.3	233	70.4	82.8	90.6
**65-**	780	616	66.6	74.8	94.5	164	54.6	64.9	88.6
**Post-menopausal**									
**No**	1,271	1,128	86.8	93.6	95.4	143	73.4	83.1	90.9
**Yes**	1,709	1,336	75.2	83.0	96.4	373	63.6	75.0	89.0
**PR status**									
**Negative**	809	326	76.0	82.0	95.4	483	66.6	77.3	89.2
**Positive**	2,171	2,138	81.2	88.7	96.0	33	61.1	76.6	93.3
**HER2 status**									
**Negative**	2,586	2,252	80.8	88.0	96.1	334	66.1	76.6	88.6
**Positive**	394	212	77.1	86.4	94.3	182	66.6	78.4	90.6
**Ki-67 status**									
**Negative**	405	392	85.5	91.2	99.7	13	64.2	77.4	-
**Positive**	1,245	1,027	79.3	88.4	95.5	218	67.4	79.4	79.1
**Unknown**	1,330	1,045	79.8	86.1	95.7	285	65.5	75.6	91.4
**Tumor size**									
**< 10 mm**	593	476	89.0	92.6	98.6	117	76.5	89.3	90.2
**10 ≤ and < 20 mm**	1,152	984	83.5	89.5	98.7	168	69.0	79.7	90.0
**20 ≤ and < 30 mm**	701	562	77.8	86.2	94.2	139	65.7	75.7	87.7
**30 ≤ and < 40 mm**	275	229	73.8	84.1	93.9	46	57.2	66.1	95.1
**40 ≤ and < 50 mm**	113	94	69.8	81.8	95.1	19	51.9	60.6	88.5
**50 mm ≤**	146	119	55.5	75.4	79.8	27	33.7	48.6	83.0
**Tumor grade**									
**1**	582	559	87.3	91.5	100	23	78.7	84.1	84.4
**2**	1,410	1,306	81.6	88.2	97.1	104	65.6	80.0	85.0
**3**	988	599	71.8	83.7	90.3	389	65.7	76.1	91.1

**Detection**									
**Screening**	1,299	1,108	84.7	90.9	97.4	191	69.4	82.5	90.1
**Symptomatic**	1,681	1,356	77.0	85.4	94.8	325	64.4	74.2	89.1
**Positive nodes**									
**0**	2,090	1,720	84.1	89.6	98.1	370	71.3	81.5	91.3
**1**	404	348	79.6	87.7	96.7	56	64.9	75.0	93.7
**2-4**	311	256	73.7	84.5	92.5	55	55.3	68.3	74.4
**5-9**	123	103	56.2	76.6	83.3	20	37.8	55.7	69.3
**10-**	52	37	35.4	61.7	66.0	15	26.7	42.8	85.1
**Surgery**									
**Mastectomy**	1,362	1,077	77.7	86.0	95.0	285	63.4	74.4	87.9
**Partial**	1,618	1,387	82.6	89.3	96.7	231	69.9	80.8	91.5
**Radiotherapy**									
**No**	1,296	1,033	80.5	86.9	96.4	263	66.9	76.9	89.2
**Yes**	1,684	1,431	80.5	88.6	95.7	253	65.6	77.7	89.6
**Hormone therapy**									
**No**	769	294	77.0	84.9	94.7	475	66.2	77.0	88.6
**Yes**	2,211	2,170	81.0	88.3	96.1	41	66.9	80.0	97.2
**Trastuzumab**									
**No**	2,716	2,312	80.7	87.9	96.1	404	66.0	77.1	88.6
**Yes**	264	152	76.6	87.2	93.2	112	67.2	77.9	91.9
**Chemotherapy**									
**No**	1,807	1,630	82.3	88.2	97.8	177	64.6	76.3	91
**2nd gen**	673	485	80.7	88.6	94.6	188	69.8	79.5	87.9
**3rd gen**	500	349	71.8	85.3	90	151	63.9	75.7	89.4
**pStage (UICC 8th)**									
**I**	1,495	1,251	85.8	90.7	98.7	244	73.2	84.2	91.7
**II**	1,029	852	78.5	86.5	95.8	177	65.5	74.9	87.8
**III**	456	361	67.0	81.3	88.1	95	50.0	63.7	86.2

Abbreviations: ER, estrogen receptor; v2.2, PREDICT version 3.0; v3.0, PREDICT version 3.0; Obs, observed survival probability by Kaplan-Meier method; PR, progesterone receptor; gen, generation; UICC, the Union for International Cancer Control
